# Supplementary material for: Suitability and user acceptance of the eResearch system “Prospective Monitoring and Management App (PIA)”—The example of an epidemiological study on infectious diseases
Source: PLoS One. 2023 Jan 3;18(1):e0279969. doi: 10.1371/journal.pone.0279969 (PMC9810156; doi:10.1371/journal.pone.0279969)
Supplement: S1 Table — Q1: First Quartile, Q3: Third Quartile. (DOCX) [file pone.0279969.s001.docx]

S1 Table. Distribution of technology readiness score (range 1 to 5) in total and across gender and age group. Q1: First Quartile, Q3: Third Quartile

|  |  | |  | Technology readiness score | | | | |
| --- | --- | --- | --- | --- | --- | --- | --- | --- |
|  |  |  | ***n*** | **Median** | **Q1** | **Q3** | **Minimum** | **Maximum** |
| Total |  |  | 253 | 2.9 | 2.7 | 3.1 | 2.0 | 4.0 |
|  |  | **Age group** |  |  |  |  |  |  |
| Gender | **Female** | **<40** | 32 | 2.7 | 2.4 | 2.8 | 2.2 | 3.3 |
|  |  | **40-60** | 67 | 2.8 | 2.88 | 3.1 | 2.0 | 3.8 |
|  |  | **>60** | 32 | 2.9 | 2.7 | 3.2 | 2.2 | 3.9 |
|  | **Male** | **<40** | 17 | 3.0 | 2.8 | 3.2 | 2.2 | 3.66 |
|  |  | **40-60** | 70 | 3.0 | 2.8 | 3.2 | 2.1 | 4.0 |
|  |  | **>60** | 33 | 3.0 | 2.8 | 3.2 | 2.2 | 3.6 |
|  | **Missing** | | 2 | - | - | - | - | - |
