# Supplementary material for: Suitability and user acceptance of the eResearch system “Prospective Monitoring and Management App (PIA)”—The example of an epidemiological study on infectious diseases
Source: PLoS One. 2023 Jan 3;18(1):e0279969. doi: 10.1371/journal.pone.0279969 (PMC9810156; doi:10.1371/journal.pone.0279969)
Supplement: S2 Table — (DOCX) [file pone.0279969.s002.docx]

S2 Table. Answers to technology readiness questionnaire (n = 258)

| Question | Total  *n* (%) | Fully agree *n* (%) | Somewhat agree *n* (%) | Neutral *n* (%) | Somewhat disagree *n* (%) | Fully disagree *n* (%) | Missing *n* (%) |
| --- | --- | --- | --- | --- | --- | --- | --- |
| I am very curious about new technical developments. | 258 (100.0) | 50 (19.4) | 98 (38.0) | 87 (33.7) | 21 (8.1) | 2 (0.8) | 0 (0.0) |
| Dealing with technical innovations is usually too much for me. | 258 (100.0) | 1 (0.4) | 10 (3.9) | 64 (24.8) | 117 (45.3) | 64 (24.8) | 2 (0.78) |
| I find it difficult to deal with new technology - most of the time I just can't. | 258 (100.0) | 1 (0.4) | 6 (2.3) | 41 (15.9) | 105 (40.7) | 103 (40.0) | 2 (0.78) |
| It is up to me whether I can use new technical developments - it has little to do with chance. | 258 (100.0) | 109 (42.2) | 106 (41.1) | 32 (12.4) | 8 (3.1) | 1 (0.4) | 2 (0.78) |
| I am always interested in using the latest technical equipment. | 258 (100.0) | 18 (7.0) | 44 (17.1) | 110 (42.6) | 64 (24.8) | 20 (7.8) | 2 (0.78) |
| When dealing with modern technology, I am often afraid that I will fail. | 258 (100.0) | 1 (0.4) | 9 (3.5) | 30 (11.6) | 89 (34.5) | 126 (48.8) | 3 (1.2) |
| If I have difficulties dealing with technology, it is ultimately up to me to solve them. | 258 (100.0) | 38 (14.7) | 91 (35.3) | 79 (30.6) | 34 (13.2) | 12 (4.7) | 4 (1.6) |
| If I had the opportunity, I would use technical products much more often than I do now. | 258 (100.0) | 14 (5.4) | 29 (11.2) | 78 (30.2) | 98 (38.0) | 35 (13.6) | 4 (1.6) |
| I'm afraid of breaking new technical developments rather than using them properly. | 258 (100.0) | 1 (0.4) | 3 (1.2) | 14 (5.4) | 80 (31.0) | 156 (60.5) | 4 (1.6) |
| What happens when I deal with new technical developments is ultimately up to my control. | 258 (100.0) | 54 (21.0) | 111 (43.0) | 70 (27.1) | 15 (5.8) | 3 (1.2) | 5 (1.9) |
| I quickly enjoy new technical developments. | 258 (100.0) | 30 (11.6) | 82 (31.8) | 103 (40.0) | 35 (13.6) | 4 (1.6) | 4 (1.6) |
| Whether or not I am successful in using modern technology largely depends on me. | 258 (100.0) | 56 (21.7) | 124 (48.1) | 58 (22.5) | 15 (15.8) | 1 (0.4) | 4 (1.6) |
