# Supplementary material for: Suitability and user acceptance of the eResearch system “Prospective Monitoring and Management App (PIA)”—The example of an epidemiological study on infectious diseases
Source: PLoS One. 2023 Jan 3;18(1):e0279969. doi: 10.1371/journal.pone.0279969 (PMC9810156; doi:10.1371/journal.pone.0279969)
Supplement: S3 Table — (DOCX) [file pone.0279969.s003.docx]

S3 Table. Answers to user acceptance questionnaire (SUS) (n=104)

| Question | Total  *n* (%) | Fully agree *n* (%) | Somewhat agree *n* (%) | Neutral *n* (%) | Somewhat disagree *n* (%) | Fully disagree *n* (%) | Missing *n* (%) |
| --- | --- | --- | --- | --- | --- | --- | --- |
| I think that I would like to use this system frequently. | 104 (100.0) | 17 (16.3) | 32 (30.8) | 40 (38.5) | 11 (10.6) | 3 (2.9) | 1 (1.0) |
| I found the system unnecessarily complex. | 104 (100.0) | 0 (0.0) | 12 (11.5) | 22 (21.2) | 36 (34.6) | 34 (32.7) | 0 (0.0) |
| I thought the system was easy to use. | 104 (100.0) | 35 (33.7) | 41 (39.4) | 16 (15.4) | 10 (9.6) | 1 (1.0) | 1 (1.0) |
| I think that I would need the support of a technical person to be able to use this system. | 104 (100.0) | 0 (0.0) | 6 (5.8) | 6 (5.8) | 17 (16.3) | 75 (72.1) | 0 (0.0) |
| I found the various functions in this system were well integrated. | 104 (100.0) | 14 (13.5) | 32 (30.8) | 40 (38.5) | 17 (16.3) | 1 (1.0) | 0 (0.0) |
| I thought there was too much inconsistency in this system. | 104 (100.0) | 1 (1.0) | 18 (17.3) | 36 (34.6) | 30 (28.8) | 18 (17.3) | 1 (1.0) |
| I would imagine that most people would learn to use this system very quickly. | 104 (100.0) | 25 (24.0) | 47 (45.2) | 22 (21.2) | 8 (7.7) | 2 (1.9) | 0 (0.0) |
| I found the system very cumbersome to use. | 104 (100.0) | 1 (1.0) | 21 (20.2) | 17 (16.3) | 33 (31.7) | 32 (30.8) | 0 (0.0) |
| I felt very confident using the system. | 104 (100.0) | 38 (36.5) | 34 (32.7) | 19 (18.3) | 9 (8.7) | 4 (3.8) | 0 (0.0) |
| I needed to learn a lot of things before I could get going with this system. | 104 (100.0) | 0 (0.0) | 4 (3.8) | 9 (8.7) | 23 (22.1) | 68 (65.4) | 0 (0.0) |
