# Supplementary material for: Suitability and user acceptance of the eResearch system “Prospective Monitoring and Management App (PIA)”—The example of an epidemiological study on infectious diseases
Source: PLoS One. 2023 Jan 3;18(1):e0279969. doi: 10.1371/journal.pone.0279969 (PMC9810156; doi:10.1371/journal.pone.0279969)
Supplement: S4 Table — CI: Confidence Interval, AIC: Akaike Information Criterion. (DOCX) [file pone.0279969.s004.docx]

S4 Table. Results for the univariate linear regression models for outcome System Usability Score. CI: Confidence Interval, AIC: Akaike Information Criterion

| Predictor |  | Estimate (95% CI) | Global *p*-value | Local *p*-value | Adjusted R^2^ | AIC |
| --- | --- | --- | --- | --- | --- | --- |
| Technology readiness score | | 10.66 (1.45 - 19.86) | - | 0.024 | 0.04 | 811.2 |
| Gender | Male | - | 0.473 | - | -0.01 | 816.0 |
|  | Female | -2.33 (-8.74 - 4.09) |  |  |  |  |
| Age group | <40 | - | 0.482 | - | -0.01 | 817.0 |
|  | 40-60 | 3.40 (-5.30 - 12.10) |  | 0.440 |  |  |
|  | >60 | 5.50 (-3.50 - 14.50) |  | 0.228 |  |  |
| App | Android | - | 0.422 | - | -0.00 | 817.6 |
|  | iOS | -1.52 (-8.93 - 5.90) |  | 0.685 |  |  |
|  | Web | -9.52 (-21.94 - 2.92) |  | 0.132 |  |  |
|  | Multiple | 1.42 (-8.18 - 11.03) |  | 0.770 |  |  |
