# Supplementary material for: Suitability and user acceptance of the eResearch system “Prospective Monitoring and Management App (PIA)”—The example of an epidemiological study on infectious diseases
Source: PLoS One. 2023 Jan 3;18(1):e0279969. doi: 10.1371/journal.pone.0279969 (PMC9810156; doi:10.1371/journal.pone.0279969)
Supplement: S5 Table — AIC: Akaike Information Criterion. *Tested against baseline model. (DOCX) [file pone.0279969.s005.docx]

S6 Table. Results for the multivariate logistic regression models for outcome compliance. AIC: Akaike Information Criterion

| Model | Formula | Log Likelihood Ratio* (*p*-value) | AIC |
| --- | --- | --- | --- |
| Univariate | Compliance ~ gender | 211.97 (<0.001) | 4926.5 |
|  | Compliance ~ age group | 279.77 (<0.001) | 4860.7 |
|  | Compliance ~ technology readiness score | 168.67 (<0.001) | 4969.8 |
|  | Compliance ~ app | 143.51 (<0.001) | 4999.0 |
|  | Compliance ~ weeks since registration | 489.28 (<0.001) | 4655.2 |
|  | Compliance ~ SUS | 895.66 (<0.001) | 4246.8 |
| Full model | Compliance ~ gender + age group + app + technology readiness score + weeks since registration | 1175.4 (<0.001) | 3983.1 |
| Full model with interaction terms | Compliance ~ gender + age group + app + technology readiness score + weeks since registration + age group * gender + technology readiness score * app + technology readiness score * age group + technology readiness * gender | 1060.1 (<0.001) | 3802.3 |
| Reduced (final) model | Compliance ~ gender + age group + technology readiness score + weeks since registration + age group * gender | 1059.5 (<0.001) | 4097.0 |

*Tested against baseline model
