# Supplementary figures and images for: Suitability and user acceptance of the eResearch system “Prospective Monitoring and Management App (PIA)”—The example of an epidemiological study on infectious diseases
Source: PLoS One. 2023 Jan 3;18(1):e0279969. doi: 10.1371/journal.pone.0279969 (PMC9810156; doi:10.1371/journal.pone.0279969)

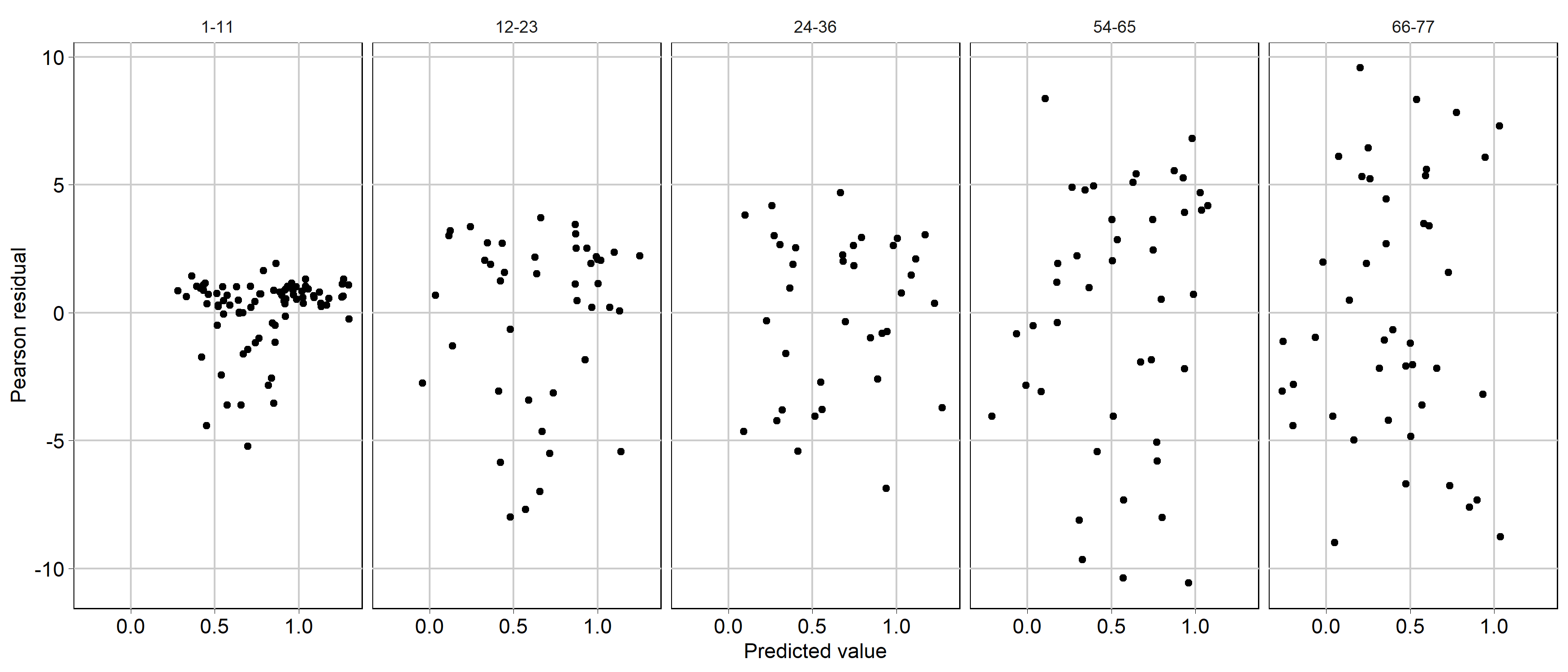

Supplement: S2 Fig — (TIFF) [file pone.0279969.s007.tiff]
