# Supplementary material for: Suitability and user acceptance of the eResearch system “Prospective Monitoring and Management App (PIA)”—The example of an epidemiological study on infectious diseases
Source: PLoS One. 2023 Jan 3;18(1):e0279969. doi: 10.1371/journal.pone.0279969 (PMC9810156; doi:10.1371/journal.pone.0279969)
Supplement: Attachment — Submitted filename: Response to Reviewers.docx [file pone.0279969.s008.docx]

**Authors' response to reviewers' comments**

| **Manuscript Number:** | PONE-D-22-02047 |
| --- | --- |
| **Authors:** | Julia Ortmann, Jana-Kristin Heise, Irina Janzen, Felix Jenniches, Yvonne Kemmling, Cornelia Frömke, Stefanie Castell |
| **Title:** | Suitability and User Acceptance of the eResearch System “Prospective Monitoring and Management App (PIA)” - the example of an epidemiological study on infectious diseases |

We sincerely thank the reviewers for their constructive feedback. We revised our manuscript accordingly and think that it improved in clarity.

| **No.** | **Reviewer #1 comments** | **Authors' responses to reviewers' comments** |
| --- | --- | --- |
|  | The authors present a clear, easy to follow analysis on the study and the topic of the study is interesting, especially given the potential of using digital tools for ongoing surveillance is a much talked about but relatively-less researched area.  In the conclusions, there are 2 major areas where I would like to see more analysis: | Thank you for your positive feedback regarding the impact of this research. |
| 1. | What inference is possible to draw here between the representativeness of the study participants who responded to user acceptance rates and the population of intended use? Since the conclusions are talking about a general use of the tool for surveillance data collection, it would be important to understand this. | The German National Cohort (NAKO), from which ZIFCO participants are recruited, is a population-based study which acquires its approximately 200.000 participants from a random sample from the registration offices. However, there is an intentional oversampling for 40+. Hence, participants in the NAKO are older than the general population on average. Additionally, selection bias - especially regarding the use of a digital application with weekly questionnaires - might play a role. Therefore, we have added a graphical comparison (newly submitted Fig 3) of the study population with the general population in Hannover, Germany, from which participants were selected in terms of age and gender [inserted in lines 110-111 and 158-161].  Also, NAKO participants who agree to use an mobile or web application for epidemiological surveys (participants who agreed to take part in ZIFCO), might be more technologically affine than the general study population.  However, syndromic monitoring including the collection of biospecimens only works digitally. Therefore, it is the only option available and any implementation and interpretation of results has to take selection bias into account; as we have added in line 278 – 281. |
| 2. | What was the reason for non-completion of questionnaires/missing information and does this have an implication on the overall conclusions? | Like taking part in NAKO, participation in the sub-cohort ZIFCO is completely voluntary. The participants can skip questions or questionnaires at any time (apart from gender and birth year in the first questionnaire) or terminate their participation. To make this clear, we stated in line 257ff: “*Earlier studies that reported high compliance with web-based questionnaires [3,31] might have had the advantage of having a fixed study period that may motivate participants to complete the study, which ZIFCO does not have. Participants in ZIFCO are asked to participate for as long as possible, ideally for years.” This is also evident in our findings that participants with shorter enrollment had better compliance than those who were enrolled for a longer period.”*  We conclude that the participants do not see their own benefit for long-term use (several years), which we aim to improve via the implementation of gamification elements into the software and more feedback functions. We stated the following:  From line 265ff*:“..*. *Integrating principles of gamification e.g. through implementation of feedback mechanisms provide an alternative to this and have been used successfully in other eHealth applications [35,36]. Adapting them is the focus of the future development in PIA and might positively influence compliance in the future particularly in younger study participants…*” |
| **No.** | **Reviewer #2 comments** |  |
|  | This paper describes acceptability and use compliance in association with an 'app' (Prospective Monitoring and Management App [PIA]) deployed to assess respiratory illness. The app was tested among participants of a German Cohort. System Usability Scale was used to assess user acceptance four months after introduction of PIA. Compliance was assessed as the proportion of weekly surveys collected over time. As well, compliance with nasal self-swabbing was assessed among participants these samples were collected from. Results show good acceptance and compliance overall. Variations by sex and age were observed. Technology readiness was a strong indicator of acceptability. The goals of the paper are well described and the methods and analyses used are appropriate. Results and data interpretation are cohesive. Overall this paper describes a potentially usable method of collecting data remotely, using app or web interface. I think the conclusions and recommendations are also good and it will be interesting to see if this can be deployed more widely. | Thank you for your positive overall assessment of the study and the manuscript. |
| 1. | Line 86: please spell what ARI refers to. | Added as suggested. |
| 2. | Line 121: was there a rationale for the threshold of 75% indicating 'good' compliance? | We added a clarification starting in line 122: “*Since there is no standard definition of good compliance in the literature, we formed quartiles and considered compliance of at least 75% as good.”* |
| 3. | Line 133 and elsewhere (eg., line 180): I am assuming that what the authors describe as 'univariate' model is actually bivariate. Please consider: Univariate statistics summarize only one variable at a time. Bivariate statistics compare two variables. | Meant are univariable (and multivariable) models including only one variable as a predictor variable, corrected in line 135, 136, 138, 185, 186, 188, 428 and 431 |
| 4. | Please describe where the N=313 comes from. It is not in the text (lines 143-148, nor in the flow chart of enrollment and data analysis. The numbers don't quite make sense. | Thank you for pointing this out. The flowchart containes now updated numbers and the corrected figure has been uploaded (Fig 1). |
